# Low-Dose Methotrexate Toxicity Presenting as Pancytopenia

**DOI:** 10.7759/cureus.32494

**Published:** 2022-12-13

**Authors:** Gundip S Dhillon, Sukhjinder Chauhan, Yema Jalal, Youssef Ghobrial, Birjees Ahmed

**Affiliations:** 1 Internal Medicine, MountainView Hospital, Las Vegas, USA; 2 Internal Medicine, Mountainview Hospital, Las Vegas, USA; 3 Internal Medicine, Sunrise Health Graduate Medical Education Consortium, MountainView Hospital, Las Vegas, USA; 4 Internal Medicine, HCA Healthcare, Nashville, USA

**Keywords:** pancytopenia secondary to low-dose methotrexate, low-dose methotrexate, granulocyte colony-stimulating factor, g-csf, poison control, sodium bicarbonate, leucovorin, methotrexate toxicity

## Abstract

High-dose methotrexate (MTX, 5 g/week) is typically used for the treatment of different malignancies and may be associated with serious side effects, such as acute kidney injury, myelosuppression, and hepatotoxicity. On the other hand, low-dose MTX (10-25 mg/week) is considered to be a safe and effective treatment for autoimmune arthropathies. Toxicity due to low-dose MTX is rare but can present with serious complications, such as pancytopenia.

In this report, we present the case of an 82-year-old woman who presented with low-dose, MTX-induced severe pancytopenia and was treated with leucovorin rescue therapy with granulocyte colony-stimulating factor (G-CSF) therapy.

## Introduction

Methotrexate (MTX) is a folic acid antagonist, which as a disease-modifying agent, has its fair share of uses ranging from treating cancer to autoimmune arthropathies. Its dose and route dictate its utility in various conditions. High-dose MTX (5 g/week) is usually used in oncology, while a lower dose (10-25 mg/week) is often used in patients with systemic immunoinflammatory rheumatological diseases [1,2].

High-dose MTX is known to cause significant toxicities, including acute kidney injury, hepatotoxicity, myelosuppression, and multiorgan failure. In contrast, low-dose MTX is considered to be relatively safe; however, it may still be associated with gastrointestinal upset, hepatotoxicity, and pulmonary damage. Nevertheless, low-dose MTX toxicity remains poorly characterized [3]. We present a unique and rare case of toxicity due to low-dose MTX. With a paucity of literature in this category to help guide clinicians, we hope to distinguish the difference between low- and high-dose toxicities through a review of the literature and a framework of pathophysiology to understand the differences and ultimately offer a management strategy for the clinician.

## Case presentation

An 82-year-old woman with a history of psoriatic arthritis was initially admitted to an outside hospital for fatigue and paresthesias. She was subsequently transferred to our hospital for oncological evaluation.

A year ago, the patient had been started on secukinumab 150 mg subcutaneously once every four weeks and MTX 20 mg/week for the treatment of psoriatic arthritis by her outpatient rheumatologist. During the COVID-19 pandemic, the patient ran out of her medications and was unable to refill them due to appointment unavailability. It was unclear how long she went without taking her medications, and then roughly four weeks before hospital admission, the patient obtained a refill and started taking MTX 20 mg/day or about 140 mg/week on her own volition (140 mg/week is still a lot less than typical high-dose 5 g/week that is used to treat malignancies), thinking that it would resolve her pain associated with arthritis faster. The patient subsequently developed gingival bleeding and presented to the outside hospital. She was found to have pancytopenia and transferred to us with concerns of MTX overdose, requiring oncologic consultation and leucovorin rescue.

Upon admission, the patient endorsed gingival bleeding, shortness of breath, and decreased urine output. She denied fevers, chills, confusion, headache, dizziness, cough, abdominal pain, nausea, vomiting, flank pain, hematuria, diarrhea, rashes, rigors, and pruritus. She was afebrile, non-tachypneic, and normotensive and had a heart rate in the range of 90 to 110 beats per minute. She was on a 3 L nasal cannula, saturating above 92%. On admission, the patient was awake, alert, and oriented. There was minimal bleeding from the gums, and no pharyngeal ulcers were noted. Pulmonary examination revealed decreased breath sounds at lung bases bilaterally, while the cardiac examination was normal. A complete blood count (CBC) panel was remarkable for pancytopenia, as demonstrated in Table 1. A chemistry metabolic panel was unremarkable. The patient’s blood urea nitrogen (BUN):creatinine (Cr) ratio was found to be 18:0.68. Vitamin B12 levels were 1313 pg/mL.

**Table 1:** Results of the CBC panel on admission. CBC, complete blood count; WBC, white blood cell; RBC, red blood cell; Hgb, hemoglobin; Hct, hematocrit; MCH, mean corpuscular hemoglobin; MCV, mean corpuscular volume; MCHC, mean corpuscular hemoglobin concentration; RDW, red cell distribution width

CBC panel	Results (reference)
WBC count	1.7 × 10^3^ microL^-1^ (4.8-10.8)
RBC count	2.77 × 10^6^ microL^-1^ (4.2-5.5)
Hgb	9.4 g/dL (12-16)
Hct	27.6% (37-47)
MCV	100 fL (80-100)
MCH	33.9 pg (27-32)
MCHC	34.1 g/dL (32-37)
RDW	11.6% (11.5-14.5)
Platelet count	90 × 10^3^ microL^-1^ (150-450)

**Figure 1:**
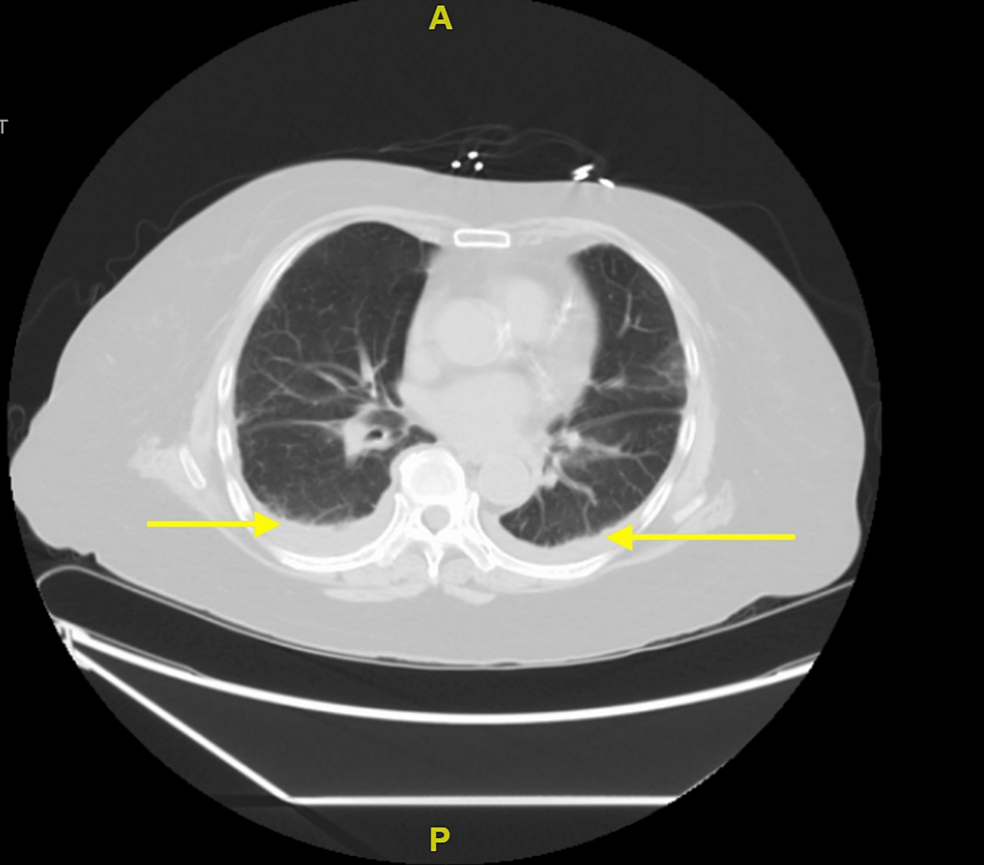
CT of the chest demonstrated small bilateral pleural effusions and possible pneumonitis, suggesting MTX toxicity. CT, computed tomography; MTX, methotrexate

A chest X-ray demonstrated reported stable cardiomediastinal silhouette, with right lower lobe linear atelectasis. Computed tomography (CT) of the chest shown demonstrated small bilateral pleural effusions and possible pneumonitis, likely caused by MTX toxicity (Figure 1).

A medical toxicologist was contacted before obtaining MTX levels who recommended initiation of leucovorin qid until the patient’s MTX level was <0.01, and sodium bicarbonate (3 amps of sodium bicarbonate) along with maintenance intravenous (IV) fluids with goal of urine pH > 7. Subsequently, the MTX levels came back as <0.04. The lab was called, and it was discovered that due to the limitation in our equipment, our hospital could only measure MTX levels up to <0.04. The medical toxicologist was contacted again, given that the patient’s levels were undetectable but she had persistent pancytopenia and no improvement in her symptoms. The medical toxicologist recommended that we utilize the normalization of pancytopenia as a marker of MTX toxicity resolution. Hematology and oncology were also consulted, and the decision was made to use granulocyte colony-stimulating factor (G-CSF) therapy to help aid the normalization of pancytopenia. After three days, the patient’s pancytopenia improved, as shown in Table 2, and her symptoms resolved. She was subsequently discharged with close follow-up in the outpatient setting with her rheumatologist.

**Table 2:** CBC panel on discharge after treatment with folinic acid. CBC, complete blood count; WBC, white blood cell; RBC, red blood cell; Hgb, hemoglobin; Hct, hematocrit; MCH, mean corpuscular hemoglobin; MCV, mean corpuscular volume; MCHC, mean corpuscular hemoglobin concentration; RDW, red cell distribution width

CBC panel	Results (reference)
WBC count	10.0 × 10^3^ microL^-1^ (4.8-10.8)
RBC count	2.56 × 10^6^ microL^-1^ (4.2-5.5)
Hgb	8.7 g/dL (12-16)
Hct	26.9% (37-47)
MCV	105 fL (80-100)
MCH	34.0 pg (27-32)
MCHC	32.3 g/dL (32-37)
RDW	13.7% (11.5-14.5)
Platelet count	115 × 10^3^ microL^-1^ (150-450)

## Discussion

High-dose MTX can result in significant toxicities, such as acute kidney injury (AKI), which is seen in 2% to 12% of patients. This occurs when MTX and its metabolites crystallize in the renal tubular lumen, resulting in tubular toxicity. Other toxicities associated with high-dose MTX include mucositis, emesis, hepatotoxicity, myelosuppression, and multiorgan failure in severe cases [4]. Despite its relative safety, low-dose MTX can also lead to toxicities, including bone marrow, hepatic, and gastrointestinal disorders. Pancytopenia constitutes approximately 1.4% of the reported adverse effects and is commonly seen in older females. The effects of pancytopenia include fatigue due to anemia, infections due to leukopenia, and bleeding secondary to thrombocytopenia. Toxicity usually starts with stomatitis and progresses to pancytopenia [5]. Hepatotoxicity, ranging from mild steatosis to cirrhosis, pulmonary damage, and gastrointestinal upset may also be seen with low-dose MTX [6].

Our patient presented with gingival bleeding, shortness of breath, pancytopenia, and a recent history of low-dose MTX use. She was also taking secukinumab for her psoriatic arthritis, which is a fully human monoclonal antibody that selectively neutralizes interleukin-17 (IL-17). Based on its mechanism of action, this medication can inhibit CD4-Th1 cells and may cause neutropenia. However, it does not suppress myeloid precursor cells and, thus, is unlikely to cause pancytopenia [7]. In addition, our patient was taking secukinumab once every four weeks for the past year, and her last dose was around one week before hospitalization. During her treatment with secukinumab, she had never developed pancytopenia before. The development of pancytopenia, gingival bleeding, and bilateral pleural effusions coinciding with a recent increase in the dose of MTX of about 20 mg/day (approximately 140 mg/week) led us to establish a diagnosis of low-dose MTX toxicity in our patient.

To prevent the complications associated with high-dose MTX therapy, monitoring plasma MTX concentration is considered essential. Plasma MTX levels are often obtained at 24, 48, and 72 hours after the initiation of MTX infusion [4]. MTX concentrations >1 µmol/L at 48 hours or >0.1 µmol/L at 72 hours are associated with an increased risk of toxicity [8]. MTX is mainly excreted by the kidneys, and therefore, renal function, including serum creatinine, BUN, urine output, and urine pH must also be assessed before, during, and after each cycle of therapy [4]. Monitoring is also recommended before and during treatment with low-dose MTX. CBC, liver function tests (LFTs), renal function tests (RFTs), and chest radiographs should be obtained before treatment initiation. CBC, LFTs, and RFTs should be assessed every 2-4 weeks for the first 3 months, every 8-12 weeks for the next 3-6 months, and every 12 weeks after that [6]. In patients who develop low-dose, MTX-induced pancytopenia, plasma MTX levels have not been found to correlate with the degree of pancytopenia [9], as was the case with our patient.

Leucovorin (folinic acid) rescue therapy is the cornerstone of high-dose MTX toxicity. It competes with MTX to enter cells and allows the replenishment of intracellular folate [10]. In patients taking long-term, low-dose MTX, 1-3 mg/week of folic acid or leucovorin may help prevent the development of adverse effects [11,12]. However, in patients who develop hematologic adverse effects or pancytopenia due to low-dose MTX toxicity, leucovorin rescue therapy with or without G-CSF therapy is recommended [10]. It is important to note that the administration of G-CSF may lead to a flare-up of the underlying arthropathy [13], which may subsequently require an increased dose of MTX. For our patient, G-CSF was only given as a one-time dose. She was also given leucovorin rescue therapy every six hours for three days. The patient responded to treatment, and her pancytopenia resolved by the fifth day of admission.

Our patient had no evidence of renal failure, based on her normal laboratory findings throughout hospitalization. Nevertheless, it must be noted that nephrotoxicity continues to be a possibility even with low-dose MTX use [14]. In such cases, the renal excretion of MTX can be enhanced by administering IV fluids to maximize urine output. In addition, MTX and its solutes are poorly soluble in acidic urine and can precipitate to cause crystalluria. Alkalinizing the urine with oral or parenteral sodium bicarbonate allows dissolution and flushing of the MTX crystals precipitated in the renal tubules, thus improving the excretion of MTX. Hemodialysis, plasma exchange, and continuous renal replacement therapy may also help increase MTX excretion [9].

## Conclusions

High-dose MTX is commonly associated with toxic effects; however, toxicity due to low-dose MTX is underreported. The purpose of this paper is to highlight the differences in the presentation and management of high-dose versus low-dose MTX overdose. Frequent laboratory monitoring is essential in establishing the diagnosis of MTX toxicity. However, it must be remembered that in high-dose toxicity, serum MTX levels dictate management, while in low-dose toxicity, drug levels do not correlate with the severity of toxicity. Leucovorin rescue therapy, G-CSF, IV hydration, and urine alkalinization are used for the treatment of low-dose MTX toxicity.
